# Effect of n-3 polyunsaturated fatty acids on the treatment of schizophrenia: an updated systematic review and meta-analysis

**DOI:** 10.1186/s12888-025-07508-6

**Published:** 2025-11-11

**Authors:** Chia-Yu Lin, Jen-Ai Lee, Tzu-Rong Peng, Pei-Yun Tsai, Pin-Hao Huang, Ming-Chia Lee, Shih Ming Chen

**Affiliations:** 1 School of Pharmacy, College of Pharmacy, Taipei Medical University, Taipei, 11031 Taiwan; 2 Department of Pharmacy, Taipei Medical University Hospital, Taipei, 11031 Taiwan; 3 Department of Pharmacy, Taipei Tzu Chi Hospital, Buddhist Tzu Chi Medical Foundation, New Taipei City, 231016 Taiwan; 4 Department of Pharmacy, New Taipei City Hospital, New Taipei City, 24141 Taiwan

**Keywords:** Schizophrenia, Ultra-high risk group, Omega-3 fatty acids, Meta-analysis, Randomized controlled trial

## Abstract

**Background:**

Schizophrenia is a chronic disorder, and treatment options for its negative and cognitive symptoms are limited. Omega-3 fatty acids have potential neuroprotective effects, but evidence of their efficacy in schizophrenia and ultra-high risk group is inconsistent. This study aimed to provide an updated meta-analysis of randomized controlled trials evaluating the effectiveness of omega-3 fatty acid supplementation in patients with schizophrenia and ultra-high risk group.

**Methods:**

A PRISMA-guided meta-analysis of randomized controlled trials comparing omega-3 fatty acids with placebo in schizophrenia was conducted. The literature search was concluded on November 12, 2024, and was conducted without any restrictions on language or timeframe. Data were extracted and analyzed using fixed or random-effects model depending on heterogeneity. Subgroup, sensitivity, and bias assessments were performed.

**Results:**

The meta-analysis, which included 16 trials with 1,435 participants, revealed no significant difference between omega-3 fatty acids and placebo in schizophrenia at the endpoint of intervention (standard mean difference = − 0.123; 95% confidence interval = − 0.267 to 0.021; *p* = 0.095), and ultra-high risk of schizophrenia at the endpoint of follow-up after intervention (standard mean difference = − 0.070; 95% confidence interval = − 0.425 to 0.285; *p* = 0.699). A small number of subgroup analyses suggested potential benefits for patients with first-episode schizophrenia and treatment over 24 weeks, and those receiving adjunctive antioxidant treatment. Publication bias was minimal.

**Conclusions:**

Omega-3 fatty acid supplementation generally has no significant benefits for treating schizophrenia and ultra-high risk group. However, subgroup findings highlight the need for future trials focusing on early-stage patients, longer supplementation, and exploring synergistic effects with adjunctive antioxidant interventions. Clinical application should remain cautious until further confirmatory evidence emerges.

**Supplementary Information:**

The online version contains supplementary material available at 10.1186/s12888-025-07508-6.

## Introduction

Schizophrenia is a chronic and severe mental disorder affecting approximately 287 in 100,000 people annually [1]. A study indicated that schizophrenia related to potential life lost with a weighted average 14·5 years [2]. Treatment of schizophrenia presents numerous challenges. Current antipsychotic medications are effective in managing its positive symptoms, such as hallucinations and delusions, but they do not alleviate its negative symptoms, including emotional flatness, lack of motivation, and cognitive impairments [3]. These residual symptoms strongly affect patients’ daily functioning and quality of life. In addition, violent crime and suicide rates are higher in patients with schizophrenia than in the general population [4]. Finally, antipsychotics frequently have side effects, such as metabolic abnormalities, weight gain, and extrapyramidal symptoms [5], that lead to poor adherence and increase the risks of relapse and hospitalization.

Schizophrenia is common in adolescents and may be related to abnormal brain structure development and continued degeneration. In brain structure studies, it has been found that ultra-high risk (UHR) and first-episode psychosis populations have lower gray matter volumes compared to healthy controls, with schizophrenia being more severe [6]. Approximately 20% to 40% of UHR cases will develop into psychosis within 3 years [7, 8].

From the neurobiological perspective, schizophrenia is strongly associated with neuro-inflammation, oxidative stress, and neurotransmitter imbalances [9]. Nutrition plays a critical role in this context because specific nutrients, such as n-3 polyunsaturated fatty acids (PUFAs), may modulate neuro-inflammation and support neuronal function, potentially affecting the disease’s progression and treatment [10, 11].

In the past, n-3 PUFAs, such as eicosapentaenoic acid (EPA) and docosahexaenoic acid (DHA), were clinically used to regulate blood lipids and reduce cardiovascular risks [12]. These fatty acids are present in phospholipids of neuronal cell membranes and improve the membranes’ fluidity and neurotransmission [13, 14]. In addition, n-3 PUFAs regulate the systems of neurotransmitters, including dopamine and serotonin, and reduce oxidative stress. n-3 PUFAs promote neuroplasticity and neurogenesis, which are crucial for brain repair and functional enhancement [15]. According to one of the theories of drug repurposing, existing drugs may acquire new indications based on their specific pharmacological effects that can provide clinical benefits for other diseases [16, 17]. Because of anti-inflammatory, neuroprotective properties, and brain cell signaling involving and modulating, n-3 PUFAs are generally considered to provide clinical benefits for psychiatric disease such as mood disorders, severe depression, bipolar depression, and relieve adverse reactions of antipsychotic drugs, such as extrapyramidal symptoms [18].

Although researchers have suggested the effectiveness of n-3 PUFAs for UHR and schizophrenia treatment [19], studies have reported conflicting results. For instance, Amminger et al. showed that n-3 PUFAs has significant clinical benefits in UHR population [20], while Winter-van Rossum et al. found no significant benefits [21]. The results of studies on schizophrenia patients are also inconsistent [22, 23]. A recent meta-analysis reported that n-3 PUFAs had non-significant clinical therapeutic effects on psychosis symptoms [24], overturning previous research results [19]. However, the studies included in that meta-analysis confounded the effects of intervention discontinuation with those of continued observation after discontinuation because it did not distinguish between these scenarios. Furthermore, the studies that continued observation were all in the UHR population, so it is necessary to clarify the effects in this population.

The present study explored whether the effect of n-3 PUFAs treatment change in schizophrenia and UHR individuals respectively, when the meta-analysis considers continued observation after n-3 PUFAs discontinuation and also investigated whether other factors affect the outcomes of this treatment.

## Methods

### Search strategy

This study adhered to the Preferred Reporting Items for Systematic Reviews and Meta-Analyses (PRISMA) guidelines [25]. Systematic searches of the PubMed, Embase, and Cochrane databases were conducted using the following keywords: [(psychosis) OR (psychotic) OR (schizophrenic) OR (schizo) OR (schizophrenia)] AND [(omega-3 fatty acids) OR (pufa) OR (eicosapentaenoic acid) OR (docosahexaenoic acid)]. The literature search was concluded on November 12, 2024, and was conducted without any restrictions on language or timeframe.

Two independent reviewers, Lin CY and Tsai PY, screened the titles and abstracts of potentially eligible studies and reviewed the full texts of relevant articles. Any discrepancies between the reviewers’ screening results were resolved with the third reviewer (Lee MC) through discussion. This study was registered in the International Prospective Register of Systematic Reviews database (registration number: CRD420251031495).

### Study selection

This meta-analysis included randomized controlled trials (RCTs) examining the efficacy of n-3 PUFAs versus placebo for patients with schizophrenia, individuals at UHR of schizophrenia, or patients having a first psychosis episode. Non-RCT studies, such as reviews, surveys, observational studies, and case reports, and studies not reporting patients’ total scores for the Positive and Negative Syndrome Scale (PANSS), Brief Psychiatric Rating Scale (BPRS), or Comprehensive Assessment of At-Risk Mental States (CAARMS) were excluded. The selection criteria were based on the Population, Intervention, Comparison, Outcome, and Study design framework and were as follows:

- Population: patients with schizophrenia or UHR of schizophrenia.
- Intervention: n-3 PUFAs.
- Comparison: placebo.
- Outcome: changes in total PANSS, BPRS, or CAARMS score.
- Study design: RCT.

### Data extraction

The following information from each RCT was extracted by two reviewers (Lin CY and Tsai PY) independently: the first author’s name, year of publication, design of the study, n-3 PUFAs dosage, diagnosis of patients, duration of intervention, type of antipsychotics, and outcome measure (total PANSS, BPRS, or CAARMS score). The efficacy of n-3 PUFAs treatment versus placebo was compared using the extracted data.

### Quality assessment

The risk of bias in each RCT was assessed by two independent reviewers (Lin CY and Tsai PY) by using version 2 of the Cochrane risk-of-bias tool for randomized trials (RoB 2) [26]. Any disagreement between reviewers was resolved through discussion with the third reviewer (Lee MC).

In RoB 2, the overall risk of bias is assessed on the basis of bias in five domains: the randomization process, deviations from the intended intervention, missing outcome data, measurement of the outcome, and selection of the reported result. For each domain, an RCT’s risk of bias was rated as high risk, low risk, or some concerns. To prevent unit-of-analysis errors caused by some RCTs having multiple arms, the control arm was split [27].

### Data synthesis and statistical analysis

Statistical analyses were performed using Comprehensive Meta-Analysis software (version 3, Biostat, Inc. Englewood, NJ, USA.). The primary outcome—change in mean total PANSS, BPRS, or CAARMS score from baseline to the end of treatment in the omega-3 fatty acid group versus placebo group—is presented as a standardized mean difference (SMD) with 95% confidence interval (CI), which were calculated using the inverse variance method. SMDs were combined using fixed model. The DerSimonian–Laird random-effects model [28], assumes significant heterogeneity among included studies, applied while heterogeneity index (*I*^*2*^) is over 50%.

Statistical heterogeneity was assessed using the heterogeneity index and corresponding p value. The degree of heterogeneity was classified as low (*I*^*2*^ = 25%–50%), moderate (*I*^*2*^ = 50%–75%), or high (*I*^*2*^ > 75%). A p value of < 0.05 was considered statistically significant. Subgroups defined by the following were analyzed for potential moderators or mediators: n-3 PUFAs dosage, n-3 PUFAs composition, treatment duration, adjunctive treatment with antioxidants, patients’ mean age, disease stage, baseline PANSS score, inpatient or outpatient, and phase of schizophrenia.

A sensitivity analysis was performed by sequentially removing each study to determine its effect on the overall effect size when I² > 50%, or I² > 25% with the analysis results were between critical values. Publication bias was evaluated using funnel-plot asymmetry, Begg and Mazumdar rank correlation, Egger’s regression, the fail-safe N test, and Duval and Tweedie’s trim-and-fill method.

## Results

### Literature search

**Fig. 1:**
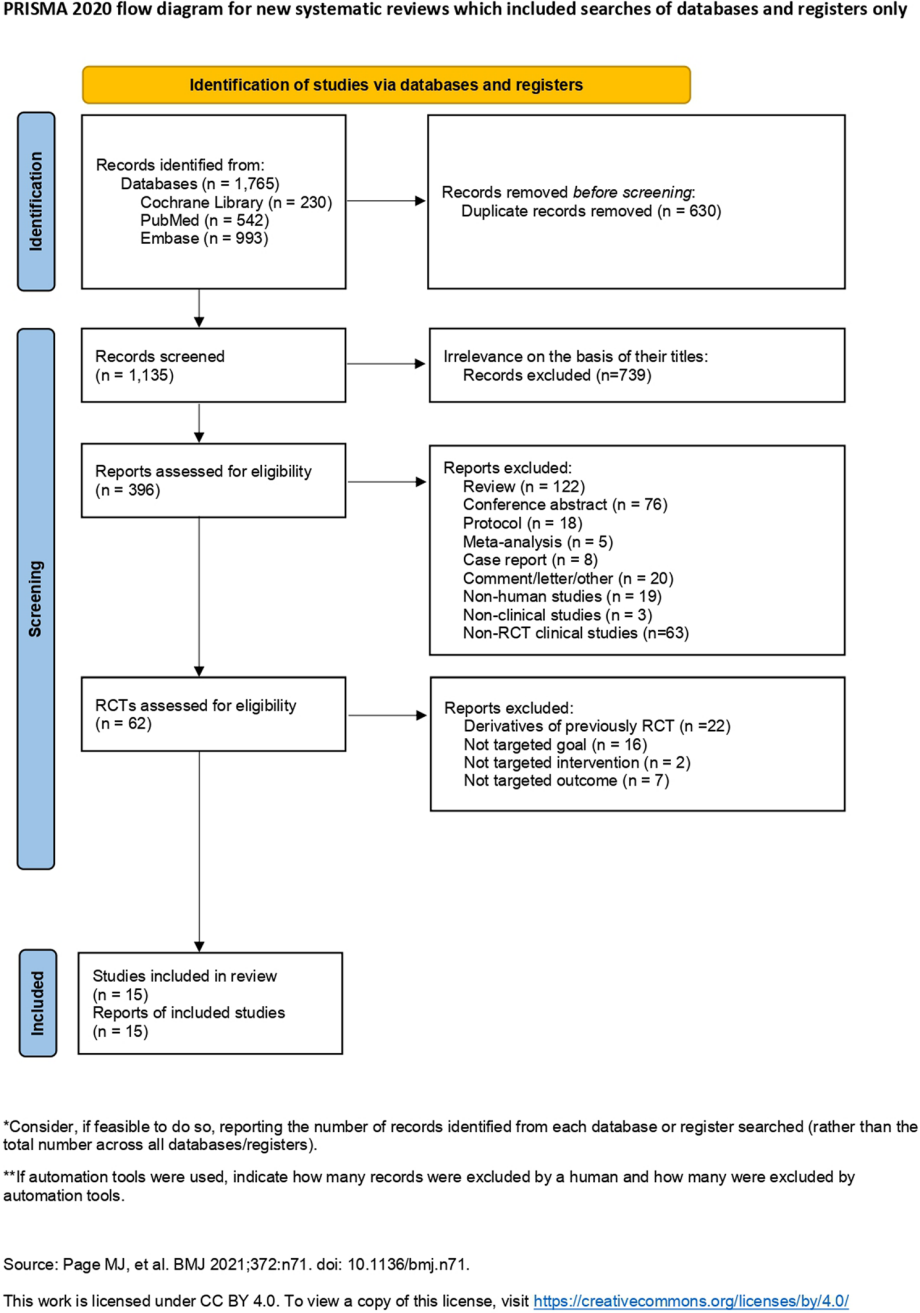
Flowchart of the systematic review and meta-analysis

The initial literature search identified 1,765 potentially eligible articles. After the removal of 630 duplicates, 1,135 articles were screened, and 739 irrelevant articles were then excluded on the basis of their title and abstract. Of 396 articles that underwent a full-text review, 62 were articles reporting an RCT and were assessed for their eligibility. A total of 46 were excluded for the following reasons: 22 were derivatives of a previously reported study, 16 did not investigate the question of interest, 2 did not involve an intervention, and 7 did not have available outcome for analysis. Ultimately, 16 RCT studies reported in 15 articles were included in the meta-analysis. The selection process is illustrated in the PRISMA flowchart displayed in Fig. 1.

### Study characteristics

The 16 RCTs included in this meta-analysis performed 18 comparisons and were conducted from 2000 to 2024. The study cohort comprised patients with a diagnosis of schizophrenia or psychosis. Of the 1,435 included patients, 728 and 707 received n-3 PUFAs and placebo, respectively.

Of the 18 included comparisons, 14 evaluated n-3 PUFAs effect on schizophrenia patients [22, 23, 29–37], whereas the other 4 on UHR individual [20, 21, 38, 39]. The n-3 PUFAs employed in the studies for the 18 comparisons were EPA or DHA (8 comparisons) or EPA + DHA (10 comparisons). The daily dose of n-3 PUFAs ranged from 300 to 3,000 mg. The duration of n-3 PUFAs treatment ranged from 6 to 104 weeks. The characteristics of the included studies are presented in Table 1. The quality assessment of the studies revealed some concerns of bias for seven studies and low risk for the other studies (Figure S1).

**Table 1:** Characteristics of the included studies

First author/year	Diagnosis	Study typology	Antioxidants (mg)	Dosage (mg/day)	Patient numbers	Adjunctive treatment	Duration of intervention (Weeks)	Follow up after intervention (weeks)	Outcomes
Fenton 2001 [19]	Schizophrenia	EPA Placebo	- -	3000 -	43 44	unspecified antipsychotics	16	-	PANSS^A^
Peet 2001−1 [20]	Schizophrenia	EPA DHA Placebo	- - -	2000 2000 -	15 16 14	unspecified antipsychotics	12	-	PANSS^A^
Peet 2001−2 [20]	Schizophrenia	EPA Placebo	- -	2000 -	14 12	Conventional antipsychotics	12	-	PANSS^A^
Emsley 2002 [21]	Schizophrenia	EPA Placebo	- -	3000 -	20 20	unspecified antipsychotics	12	-	PANSS^A^
Manteghiy 2008 [22]	Schizophrenia	EPA + DHA Placebo	- -	1080 + 720 -	42 43	Risperidone	6	-	PANSS^A^
Amminger 2010 [31]	Ultra-high risk	EPA + DHA Placebo	Vit E (7.6) Vit E (7.6)	700 + 480 -	34 33	Monotherapy	12	40	PANSS^B^
Bentsen 2013 [23]	Schizophrenia	EPA Placebo EPA Placebo	Vit C/E (1000/368) Vit C/E (1000/364) Vit E (4) -	2000 - 2000 -	18 28 33 25	unspecified antipsychotics	16	-	PANSS^A^
Jamilian 2014 [24]	Schizophrenia	EPA Placebo	- -	300 -	30 30	atypical antipsychotics	8	-	PANSS^A^
Pawełczyk 2016 [25]	First-episode schizophrenia	EPA + DHA Placebo	Vit E 0.2% Vit E 0.2%	1320 + 880 -	36 35	unspecified antipsychotics	26	-	PANSS^A^
McGorry 2017 [26]	Ultra-high risk	EPA + DHA Placebo	- -	840 + 560 -	153 151	with CBCM	26	26	BPRS^A, B^
Qiao 2018 [27]	Schizophrenia	EPA + DHA Placebo	- Vit E (10)	540 + 360 -	28 22	unspecified antipsychotics	12	-	PANSS^A^
Robinson 2019 [28]	Schizophrenia	EPA + DHA Placebo	Vit E (4) -	740 + 400 -	25 25	Risperidone	16	-	BPRS^A^
Qiao 2020 [29]	Schizophrenia	EPA + DHA Placebo	- Vit E (10)	540 + 360 -	32 35	± Clozapine /Olanzapine	8	-	PANSS^A^
Tang 2020 [30]	Schizophrenia	EPA + DHA Placebo	- Vit E (10)	720 + 480 -	40 40	Olanzapine	12	-	PANSS^A^
Qurashi 2024 [32]	Ultra-high risk	EPA + DHA Placebo	- -	720 + 480 -	82 82	Monotherapy	26	26	CAARMS^B^
Winter-van Rossum 2024 [13]	Ultra-high risk	EPA + DHA Placebo	Vit E (7.6) -	720 + 480 -	67 68	Risperidone	26	78	PANSS^A, ^

*CBCM* Cognitive behavioral case management, *PANSS* The Positive and Negative Syndrome Scale, *BPRS* Brief Psychiatric Rating Scale, *CAARMS* Comprehensive Assessment of the At-Risk Mental States

^A^available outcome data at the endpoint of intervention

^B^Available outcome data at the endpoint of follow up after intervention

### Efficacy of n-3 PUFAs against schizophrenia or UHR group

**Fig. 2:**
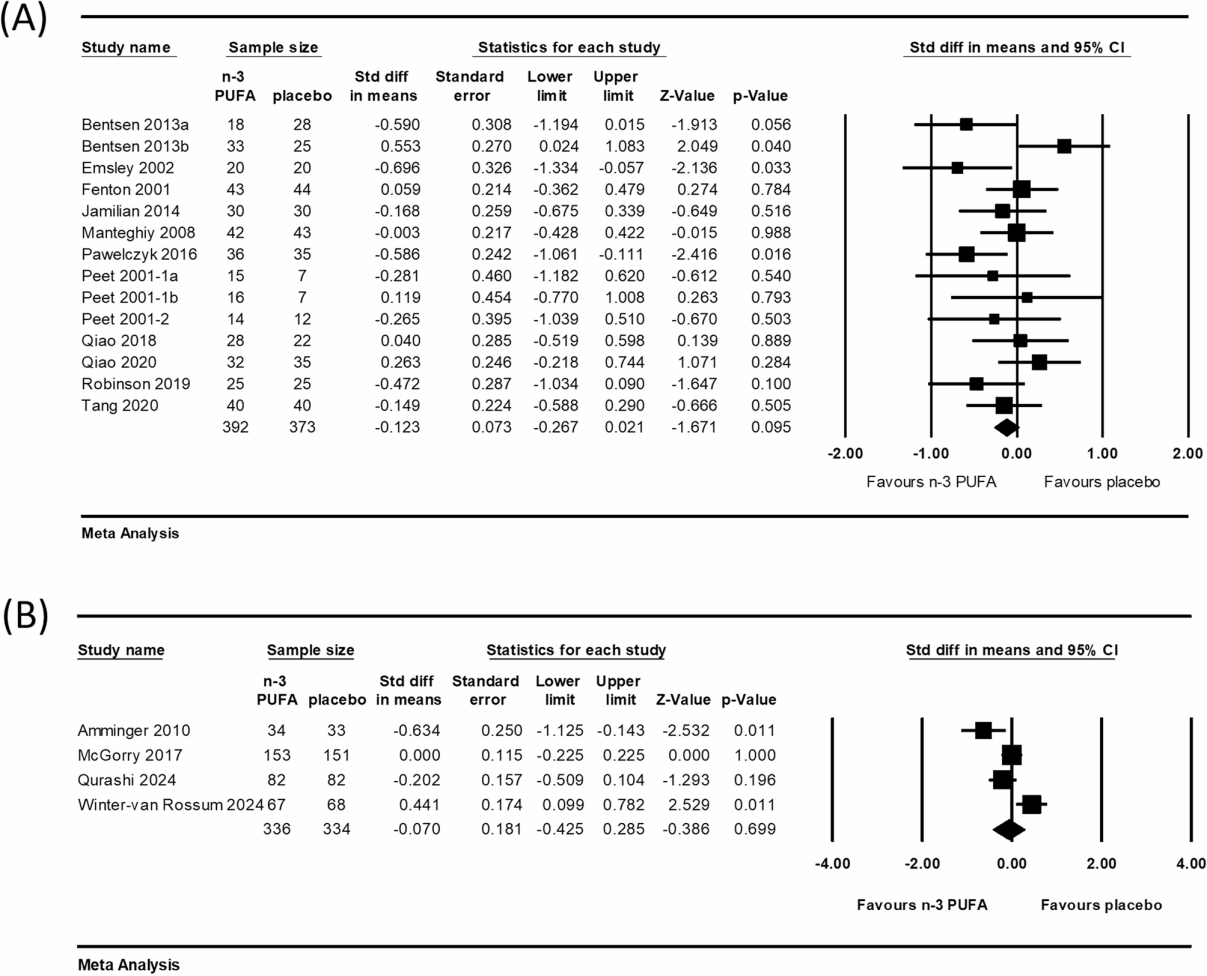
Two meta-analyses of n-3 fatty acid versus placebo on the treatment changes in mean PANSS, BPRS, or CAARMS. **A** Score changes in mean from baseline to the endpoint of intervention of schizophrenia patients; (**B**) Score changes in mean from baseline to the endpoint of follow-up after intervention of UHR group

The efficacy at the intervention endpoint of n-3 PUFAs against schizophrenia, the severity of which was measured through the PANSS or BPRS, was determined in 14 comparisons. No significant difference was noted between the n-3 PUFAs and placebo groups in terms of the mean PANSS or BPRS score change from the baseline to the conclusion of the treatment (SMD = − 0.123; 95% CI = − 0.267 to 0.021; *I*^*2*^ = 38.61%; *p* = 0.095; Fig. 2A).

The outcome at the end of a long-term follow-up after intervention discontinuation, also the UHR group, was evaluated in the 4 other comparisons. Meta-analysis of these 4 comparisons revealed no significant difference between the n-3 PUFAs and placebo groups in terms of the change in mean PANSS or CAARMS score from baseline to the end of the follow-up (SMD = − 0.070; 95% CI = − 0.425 to 0.285; *I*^*2*^ = 79.15%; *p* = 0.699; Fig. 2B).

### Subgroup analysis

The outcome was more favorable in the n-3 PUFAs group than in the placebo group for the subgroup analyses based on adjunctive treatment with antioxidants (2 RCTs, *N* = 117; SMD = − 0.587; 95% CI: −0.961 to − 0.214; *p* = 0.002), the stage of first-episode schizophrenia and the treatment duration over 24 weeks with the same one study (1 RCT, *N* = 71; SMD = − 0.586; 95% CI: −1.061 to − 0.111; *p* = 0.016). No significant differences were found in the subgroup analyses based on n-3 PUFAs dosage, n-3 PUFAs composition, treatment duration, mean age, baseline PANSS, inpatient or outpatient, and the phase of schizophrenia (Table 2).

**Table 2:** Significant amelioration of schizophrenia by n-3 PUFAs in subgroup analysis

Subgroup	RCTs	N	SMD	Lower limit	Upper limit	*p* Value
Dosage of n-3 PUFA						
< 1000 mg	2	117	0.168	−0.197	0.532	0.367
1000~2000 mg	3	198	−0.122	−0.348	0.104	0.290
> 2000 mg	9	450	−0.374	−0.863	0.115	0.134
Composition of n-3 PUFA						
DHA only	1	22	−0.281	−1.182	0.620	0.540
EPA only	7	340	−0.124	−0.452	0.203	0.457
EPA+DHA	6	403	−0.144	−0.398	0.109	0.265
Treatment duration						
< 12 weeks	3	212	0.034	−0.236	0.304	0.806
12~24 weeks	10	482	−0.126	−0.308	0.056	0.176
≥ 24 weeks	1	71	−0.586	−1.061	−0.111	0.016
Anti-oxidant						
With anti-oxidant	2	117	−0.587	−0.961	−0.214	0.002
Without anti-oxidant	6	256	−0.188	−0.438	0.062	0.140
Imbalance to analyze	6	392	-	-	-	-
Mean age						
20~30	5	305	−0.243	−0.656	0.170	0.249
>30	9	460	−0.049	−0.234	0.136	0.606
Stages						
First-episode psychosis	1	71	−0.586	−1.061	−0.111	0.016
Schizophrenia	13	694	−0.090	−0.274	0.095	0.341
Baseline PANSS						
<60	1	80	−0.149	−0.588	0.290	0.505
60~90	10	469	−0.064	−0.249	0.120	0.495
>90	3	216	−0.236	−0.504	0.033	0.085
Clinical setting						
Inpatient	6	377	−0.042	−0.247	0.163	0.690
Outpatient	8	388	−0.201	−0.403	0.001	0.051
Phase of schizophrenia						
Acute phase	5	264	−0.195	−0.439	0.049	0.118
Stable phase	5	252	−0.148	−0.400	0.103	0.247
Undefined	4	249	−0.020	−0.272	0.232	0.878

*CBCM* Cognitive behavioral case management, *PANSS* The Positive and Negative Syndrome Scale

### Publication bias

**Fig. 3:**
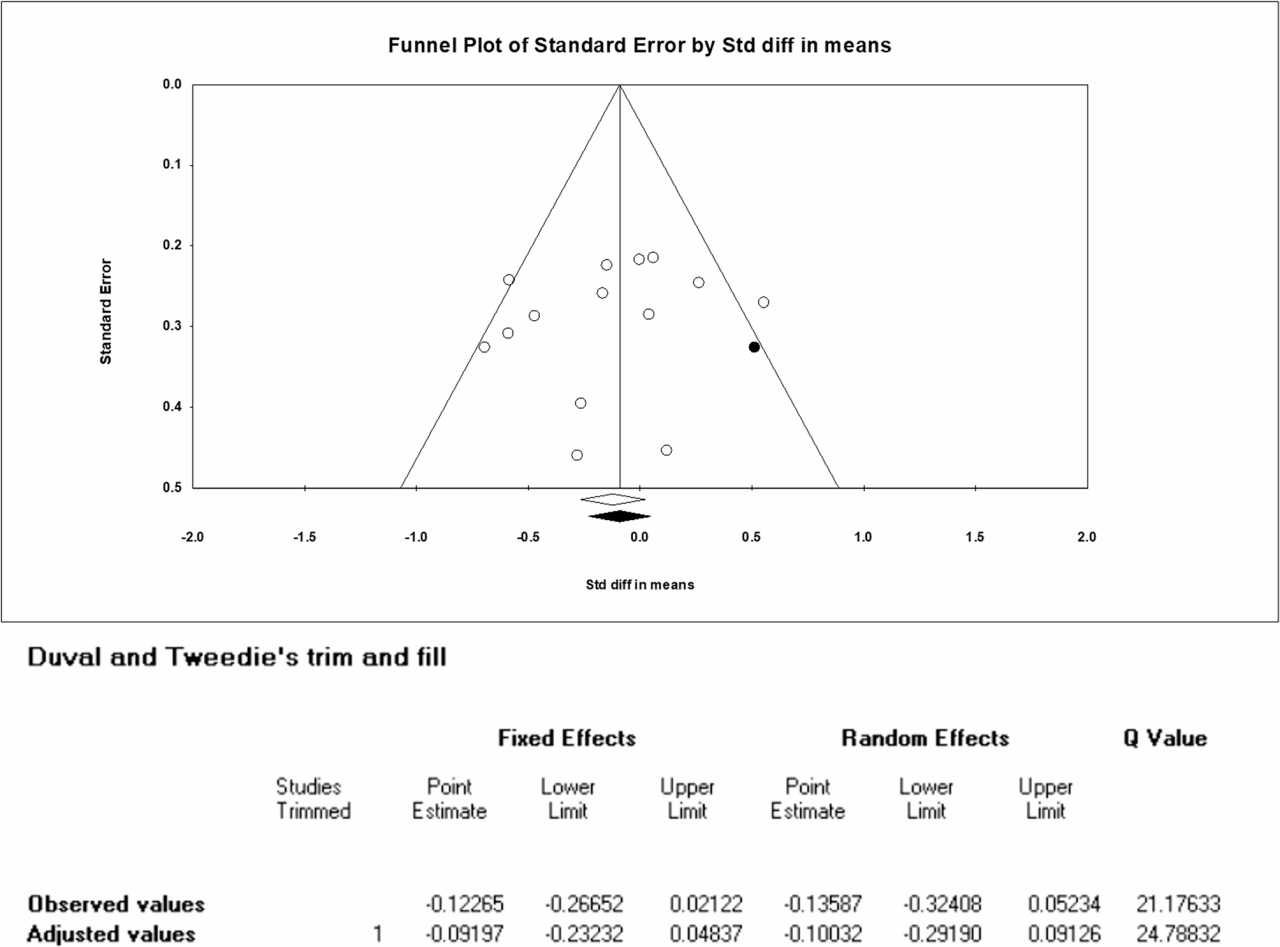
Funnel plot depicting publication bias: n-3 PUFAs versus placebo of schizophrenia patients

Publication bias was assessed for the 14 comparisons of schizophrenia group. Asymmetry was detected by visually inspecting funnel plots (Fig. 3). One spot needed to be added on the right side through trim-and-fill correction (adjusted SMD = −0.092, CI: −0.232 to 0.048). Egger test and Begg test revealed no significant publication bias in the schizophrenia group (two-tailed *p* = 0.452 and 0.228, respectively). The same results were also shown in UHR group. (two-tailed *p* = 0.640 and 0.734, respectively).

### Sensitivity analysis

**Fig. 4:**
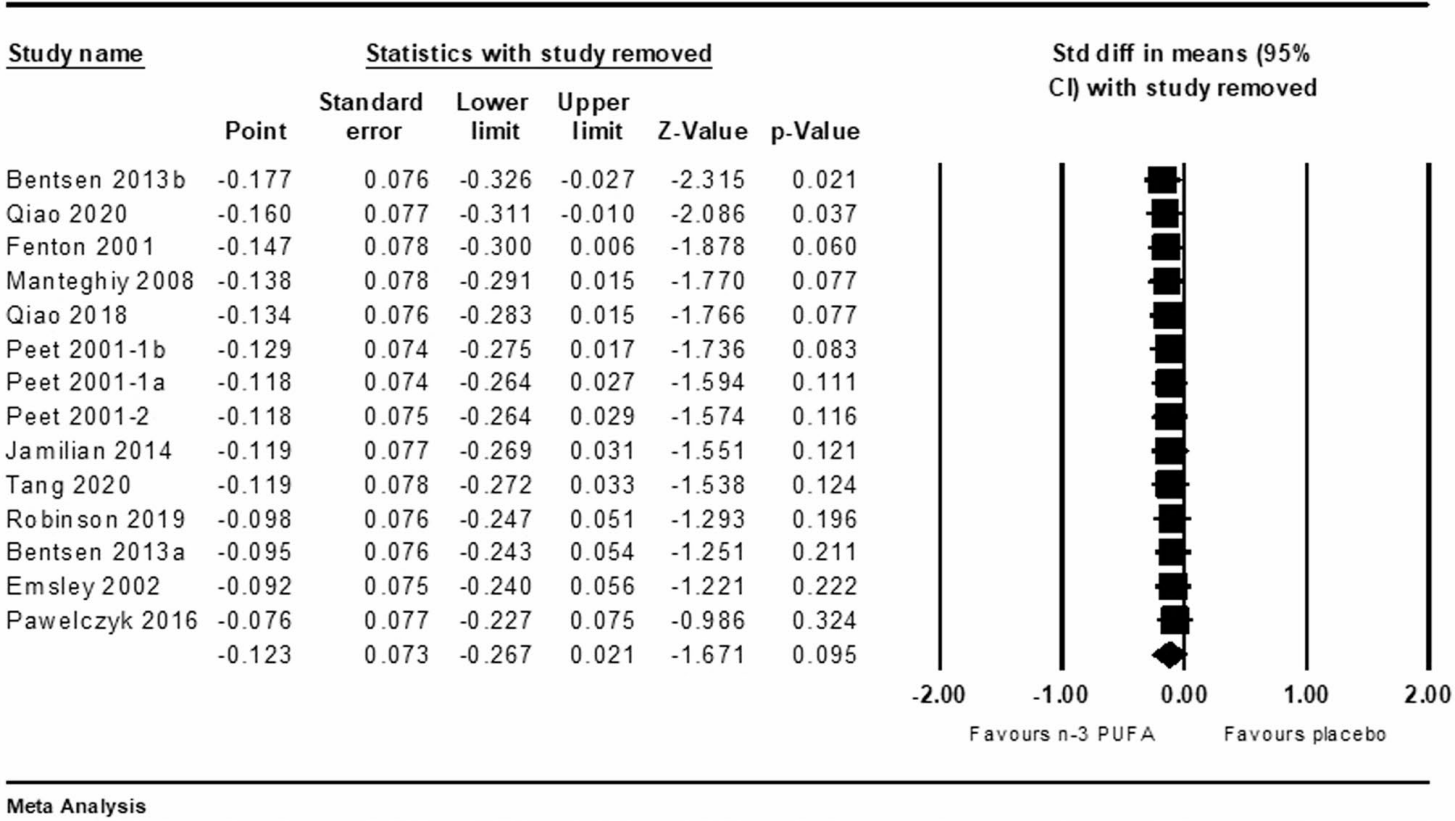
Sensitivity analysis for n-3 PUFAs versus placebo of schizophrenia patients

Sensitivity analysis was performed using the leave-one-out method to evaluate the robustness of the meta-analysis results. For the analysis of schizophrenia, the pooled SMD ranged from − 0.177 to − 0.076, and the lower and upper bounds of the 95% CI value ranged from − 0.326 to − 0.267 and − 0.027 to 0.021 respectively. The removal of Bentsen 2013b or Qiao 2020 may decrease heterogeneity and affect the overall findings (Fig. 4). For the analysis of UHR group, the pooled SMD ranged from − 0.217 to 0.068, and the lower and upper bounds of the 95% CI value ranged from − 0.687 to − 0.261 and 0.095 to 0.461 respectively (Figure S3).

## Discussion

The results of the present study indicate no significant benefits of n-3 PUFAs use in schizophrenia treatment, which is different from the finding of Goh et al. [19] but consistent with that of Rossier and Hallahan [24]. The possible reason is that we analyzed studies with different endpoint (intervention discontinuation or continued follow-up) separately and also clarified the differences between the schizophrenia and UHR populations. In the schizophrenia group, we separated the UHR studies to reduce heterogeneity to the level suitable for a fixed model, which is more reasonable than previous research applying fixed model for high heterogeneity analysis [19]. On the other hand, previous researches [19, 24] have not separate out the studies in which the outcome was measured 6–9 months after the discontinuation of n-3 PUFAs. Therefore, we extracted studies with the endpoint of follow-up data in UHR individual from the endpoint of intervention discontinuation in schizophrenia group. And the results of our both analyses indicated no significant differences between n-3 PUFAs and placebo in schizophrenia and UHR treatment.

In UHR studies, researchers typically aim to evaluate the medium to long-term benefits of interventions and their potential to reduce the risk of psychotic transition. However, 4 RCTs included in the present analysis that monitor patients for 6–9 months after n-3 PUFAs discontinuation, a period that far exceeds the biological half-life of n-3 PUFAs [40, 41]. Consequently, the benefits of n-3 PUFAs supplementation may wane over time, while the influence of other factors may become more prominent during this follow-up period. Therefore, we divided our analysis into two groups to better distinguish differences in endpoint definitions and study populations.

Amminger et al. reported that the significant effects of n-3 PUFAs in ultra-high risk (UHR) population could have lasted for several years after 12-week intervention [20, 42]. However, subsequent studies included UHR participants did not obtain the expected results. McGorry et al. [38] and Qurashi et al. [39] reported no significant benefit of n-3 PUFAs supplement, and Winter-van Rossem et al. [21] even discovered a more favorable trend in the placebo group. This may be related to differences in study design, such as the structure of the inclusion population, dosage, concurrent medications, or non-pharmacological treatment. Improved treatment is also an important factor, Formica et al. conducted a retrospective survey of 105 UHR cases from 1995 to 2006. They found that increased frequency of cognitive behavioral therapy (CBT) was significantly associated with a reduced risk of psychosis in the UHR group [43].

The NEURAPRO study by McGorry et al., the only one included in present analysis that explicitly used cognitive behavioral case management (CBCM) as an adjunctive therapy, echoes this view that non-pharmacological interventions may have a ceiling effect on the treatment of UHR [38, 44]. In addition, the ceiling effect may also have originated from the progress of pharmacotherapy and the maturity of the included patients, which could have reduced the opportunity for n-3 PUFAs to have an effect [45].

In the present subgroup analyses for schizophrenia group, significant treatment benefits of n-3 PUFAs were found only for the subgroups based on adjunctive treatment with antioxidants, first-episode psychosis and treatment over 24 weeks, and the included RCTs were few for these subgroups.

The follow-up study of the NEURAPRO trial reported that the n-3 PUFAs concentrations in erythrocyte membranes were significantly inversely correlated with the severity of psychotic symptoms [46]. This concentration may thus serve as a potential biomarker of recovery from mental illness or indicate whether n-3 PUFAs supplementation is necessary. A recent observational analysis determined that the concentrations of EPA and DHA in the erythrocyte membranes of patients with an at-risk mental state or a first-episode of schizophrenia were lower than those in healthy controls [47]. Another study indicated that in the early stages of antipsychotic treatment for schizophrenia, the concentration of n-3 PUFAs in erythrocyte membranes can reflect the therapeutic effect of the antipsychotic [48]. Nonetheless, further studies are needed to confirm whether correcting the n-3 PUFAs level in erythrocyte membranes may improve psychotic symptoms.

The n-3 PUFAs, especially DHA, are major components of phospholipids in brain and nerve cells. A study reported that n-3 PUFAs have neuroprotective effects because they enhance the fluidity of membranes, regulate the activity of membrane-bound receptors, and help maintain normal neural signal transmission [49]. Additionally, n-3 PUFAs are considered antioxidants because of their anti-inflammatory effects, including inhibition of leukocyte chemotaxis, adhesion, and the production of pro-inflammatory cytokines such as prostaglandins and leukotrienes, which is achieved through the n-6 fatty acid metabolic pathway [50].

Antioxidant supplementation has long been considered a means of treating schizophrenia. One study found that vitamin C improved the BPRS scores of patients with schizophrenia [51]. Das et al. reported that the glutathione concentration in the anterior cingulate cortex was significantly lower in patients with schizophrenia than in healthy controls, suggesting poor protection against oxidation in schizophrenia [52].

Of the studies included in our subgroup analysis of adjunctive treatment with antioxidants, the study by Bentsen et al. [32] used higher doses of antioxidants (vitamin C and E) instead of used as a stabilizer (low dose vitamin E) for fish oil, which were employed in other studies. Bentsen et al. indicated that the worsen psychosis is related to oxidative stress. Insufficient overall antioxidant capacity will cause vitamin E and n-3 PUFAs to be oxidized and lose neuroprotective effects. Vitamin C supplementation can improve the antioxidant capacity [51] and maintain the effect of n-3 PUFAs [32].

However, a trial using antioxidants [53] that was included in two other meta-analyses [19, 24] was excluded from our analysis. In the trial, alpha lipoic acid was used as a bioactive antioxidant plus n-3 PUFAs in the experimental group. The subject of the experiment design was not solely n-3 PUFAs, and the trial thus did not meet our inclusion criteria, although the results did not reveal a significant therapeutic effect. A recent RCT obtained a similar result—no significant effect of alpha lipoic acid as an adjunctive treatment for schizophrenia [54].

Furthermore, in our subgroup analysis, 26 weeks of n-3 PUFAs treatment was associated with significant benefits in patients with the first-episode schizophrenia [34]. Although this finding is based on a single study (*N* = 71), it is consistent with previous reports showing lower level of n-3 PUFAs in the erythrocyte membranes of patients with first-episode schizophrenia [47]. According to a pharmacokinetic study, after ingestion of n-3 PUFAs, EPA and DHA level in erythrocyte membranes reached approximately half of their peak levels by day 28 and attained peak levels by day 180 [55]. Therefore, extending n-3 PUFAs treatment to 26 weeks may be reasonable, but this recommendation should be interpreted with caution and warrants further investigation in future trials.

The present study had more rigorous inclusion criteria than other analyses did and clarified details that have not been adequately addressed previously. This study determined whether the discontinuation of medication or continued follow-up affects the impact of n-3 PUFAs on schizophrenia and UHR individual, and possible influencing factors, such as the use of antioxidants, and the first-episode schizophrenia with long-term intervention.

The present study has several limitations. First, four of the included RCTs of UHR individual [20, 21, 38, 39] used a follow-up outcome in analysis, and few RCTs could be included in the subgroup analyses of schizophrenia for which significant differences were obtained. The low number of studies meant that the pooled analysis had low statistical power, potentially resulting in high uncertainty regarding the estimated effect. In addition, the present analysis is likely to have publication bias. We estimated that one study with negative results should be filled using Duval and Tweedie’s trim-and-fill method, and the adjusted result would be more favorable to the placebo group. Nonetheless, this does not affect the inference of the present analysis. Furthermore, high heterogeneity was observed in the UHR analysis comprising four RCTs. However, the overall finding of no significant benefit from n-3 PUFAs persisted even after sensitivity testing with the one-study removal method. The schizophrenia analysis included 14 comparisons showed moderate heterogeneity. Sensitivity testing identified two studies that contributed most to the heterogeneity. However, these studies did not present major methodological concerns and were therefore retained in the analysis.

## Conclusion

In summary, under most conditions, n-3 PUFAs supplementation has not been reported to have significant benefits in the treatment of schizophrenia and UHR individual. Subgroup analyses suggest that n-3 PUFAs may offer particular benefits for patients with first-episode schizophrenia receiving treatment for more than 24 weeks, as well as in combination with adjunctive antioxidants. Although the number of studies included in the subgroup analysis is limited, resulting in insufficient strength of evidence, the findings still provide directions for further exploration and targeted investigation in future research. Cautious clinical application of n-3 PUFAs is warranted until future research establishes their efficacy and delineates suitable patient populations.

## Search on November 12^th^ 2024

### Cochrane

MeSH terms

#1“omega-3 fatty acids”= 6,064

#2“pufa”= 2,376

#3“Eicosapentaenoic acid”=3,303

#4“Docosahexaenoic Acid”=3,711

#5“schizophrenia”= 21,330

#6“schizo”=135

#7“schizophrenic”= 5,359

#8“psychotic”= 9,490

#9“psychosis”= 8,600

#10(omega-3 fatty acids) OR (pufa) OR (eicosapentaenoic acid) OR (Docosahexaenoic Acid)= 9,536

#11(psychosis) OR (psychotic) OR (schizophrenic) OR (schizo) OR (schizophrenia) = 28,785

#12((psychosis) OR (psychotic) OR (schizophrenic) OR (schizo) OR (schizophrenia)) AND ((omega-3 fatty acids) OR (pufa) OR (eicosapentaenoic acid) OR (docosahexaenoic acid))= 231

### Pubmed

MeSH terms

#1 “omega-3 fatty acids”= 37,856

#2 “pufa”= 16,042

#3 “eicosapentaenoic acid”= 14,530

#4“docosahexaenoic acid”= 19,978

#5“schizophrenia”= 170,427

#6“schizo”=559

#7“schizophrenic”= 56,484

#8“psychotic”= 83,085

#9“psychosis”= 102,663

#10(omega-3 fatty acids) OR (pufa) OR (eicosapentaenoic acid) OR (docosahexaenoic acid)= 51,657

#11(psychosis) OR (psychotic) OR (schizophrenic) OR (schizo) OR (schizophrenia) = 244,222

#12((psychosis) OR (psychotic) OR (schizophrenic) OR (schizo) OR (schizophrenia)) AND ((omega-3 fatty acids) OR (pufa) OR (eicosapentaenoic acid) OR (docosahexaenoic acid))= 542

### Embase

MeSH terms

#1“omega-3 fatty acids”= 30,421

#2“pufa”= 19,789

#3“eicosapentaenoic acid”= 16,129

#4“docosahexaenoic acid”= 32,241

#5“schizophrenia”= 267,549

#6“schizo”= 1,060

#7“schizophrenic”= 37,153

#8“psychotic”= 70,164

#9“psychosis”= 171,422

#10(omega-3 fatty acids) OR (pufa) OR (eicosapentaenoic acid) OR (docosahexaenoic acid)= 64,684

#11(psychosis) OR (psychotic) OR (schizophrenic) OR (schizo) OR (schizophrenia) = 381,208

#12((psychosis) OR (psychotic) OR (schizophrenic) OR (schizo) OR (schizophrenia)) AND ((omega-3 fatty acids) OR (pufa) OR (eicosapentaenoic acid) OR (docosahexaenoic acid))= 993

## Supplementary Information

Supplementary Material 1.

## Data Availability

All data, models, and code generated or used during the study appear in the submitted article.
